# Supplementary material for: A spatial long-read approach at near-single-cell resolution reveals developmental regulation of splicing and polyadenylation sites in distinct cortical layers and cell types
Source: Nat Commun. 2025 Aug 29;16:8093. doi: 10.1038/s41467-025-63301-9 (PMC12397408; doi:10.1038/s41467-025-63301-9)
Supplement: Supplementary file 1 — Supplementary Information [file 41467_2025_63301_MOESM1_ESM.pdf]

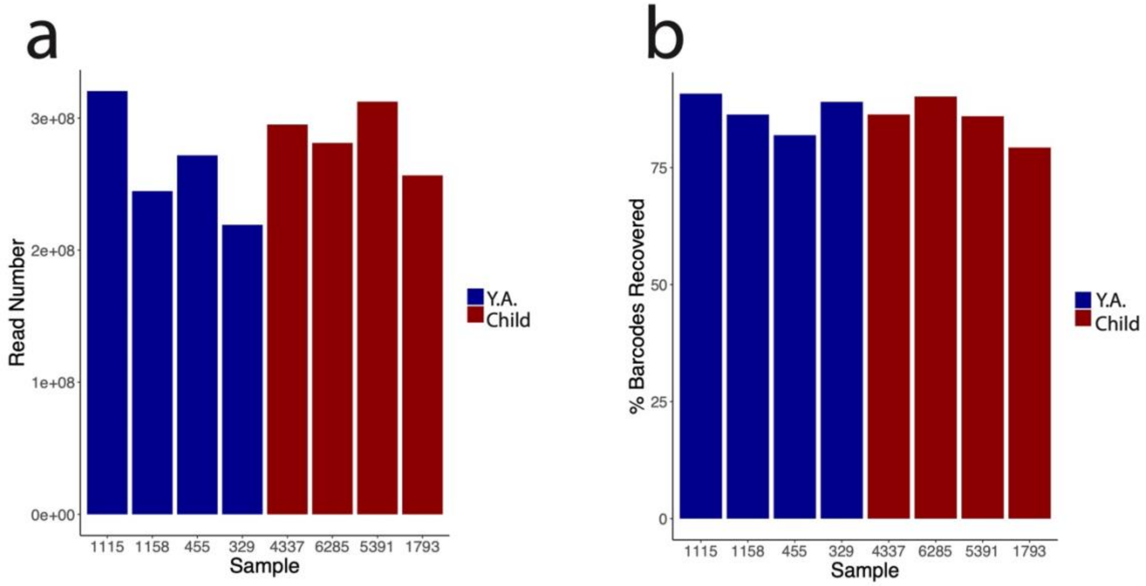

Figure S1 Illumina Data Overview. **a)** Number of sequenced Illumina reads by sample. **b)** Percent of barcodes recovered from Illumina data. Blue indicates Y.A. age group and red indicates Child age group.

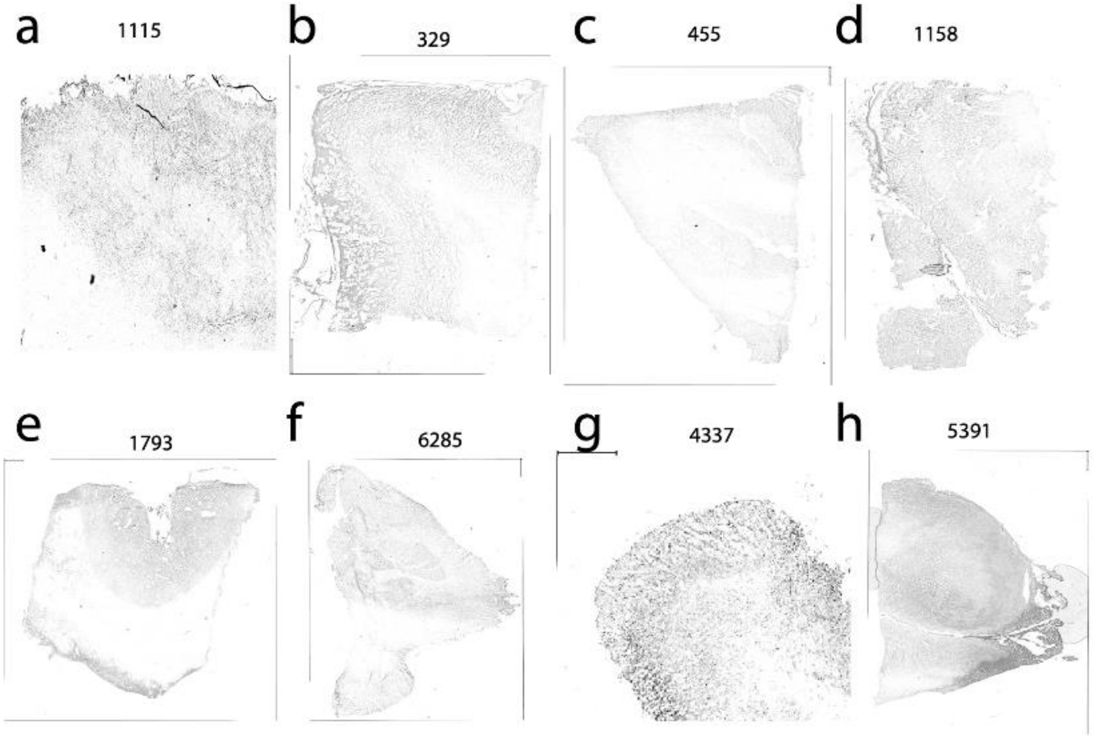

Figure S2 H&E Stains. **a-h)** Hematoxylin and Eosin staining of 10 uM tissue slice following experimental slice for each sample. Panels a-d are in the young adult group. Panels e-h are in the child group.

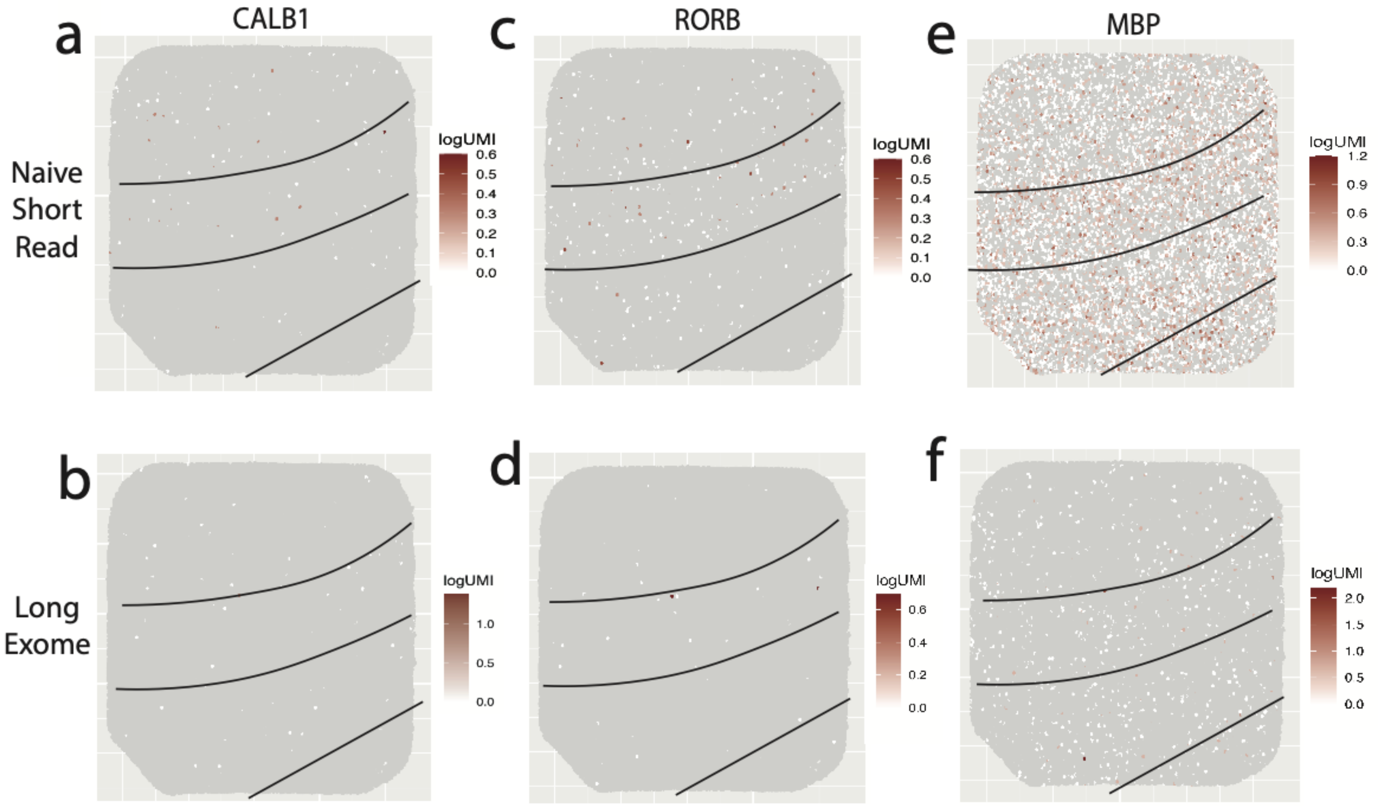

Figure S3 Marker Gene Expression. **a)** Log10(UMI) counts of Naïve Short-Read data per spot of gene CALB1 plotted by spatial location. **b)** Log10(UMI) counts of Long-Exome LR data per spot of gene CALB1 plotted by spatial location. **c)** Log10(UMI) counts of Naïve Short-Read data per spot of gene RORB plotted by spatial location. **d)** Log10(UMI) counts of Long-Exome LR data per spot of gene RORB plotted by spatial location. **e)** Log10(UMI) counts of Naïve Short-Read data per spot of gene MBP plotted by spatial location. **f)** Log10(UMI) counts of Long-Exome LR data per spot of gene MBP plotted by spatial location.

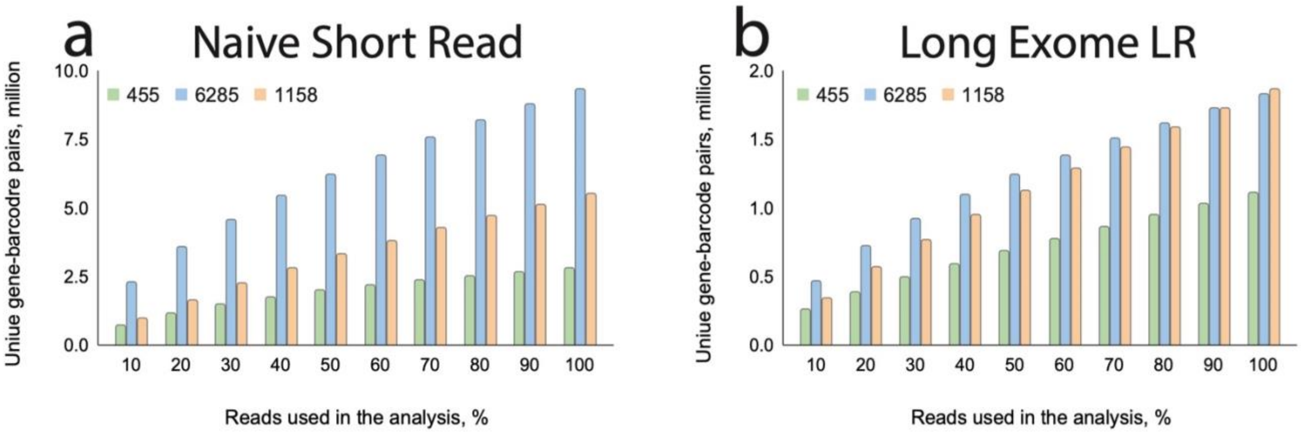

Figure S4 Gene-Barcode Pairs. **a)** Unique gene-barcode pairs in Naive SR data plotted in bins per fraction of data processed. **b)** Unique gene-barcode pairs in Long Exome LR data plotted in bins per fraction of data processed. Color indicates 3 individual samples.

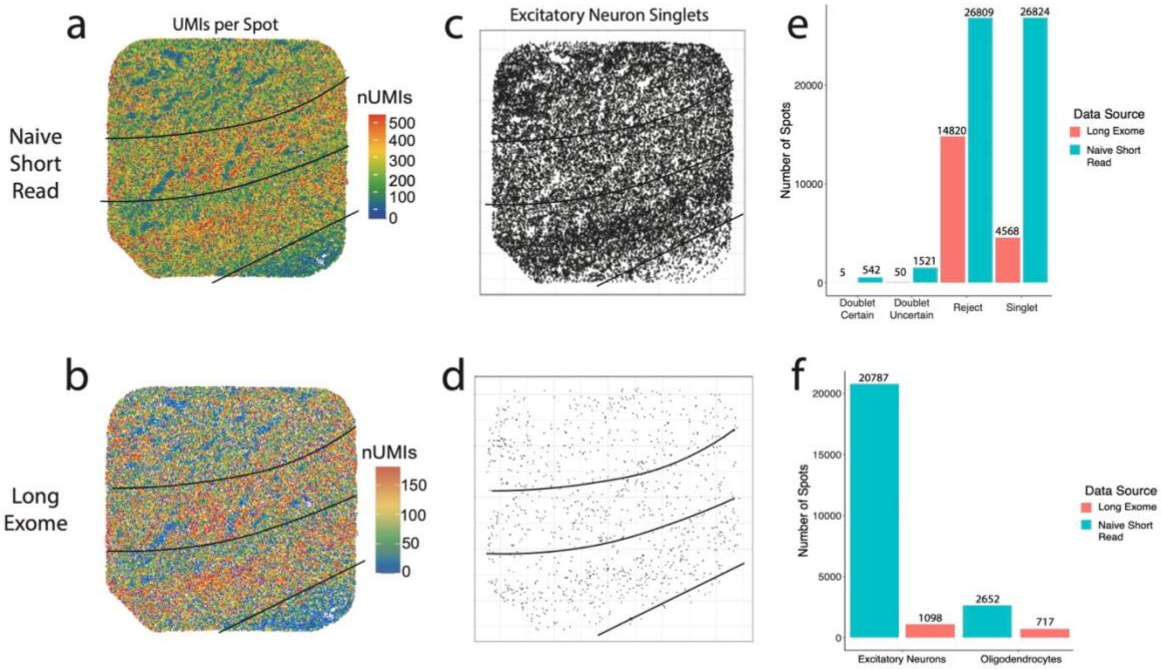

Figure S5 Cell Type Deconvolution from Short and Long reads. **a)** UMIs per spot from Naive short-read data plotted by spatial location. **b)** UMIs per spot from Long-Exome data plotted by spatial location. **c)** Excitatory-neuron singlets and their positions identified from inputting Naive Short-Read data into a deconvolution program. **d)** Excitatory-neuron singlets and their positions identified from inputting Long-Exome data into a deconvolution program. **e)** Number of identified "Doublet Certain", "Doublet Uncertain", "Reject", or "Singlet" spots by data input. **f)** Number of identified excitatory-neuron and oligodendrocyte singlets by data input. Red indicates data from Long Exome and blue indicates data from Naïve Short Reads in e,f.

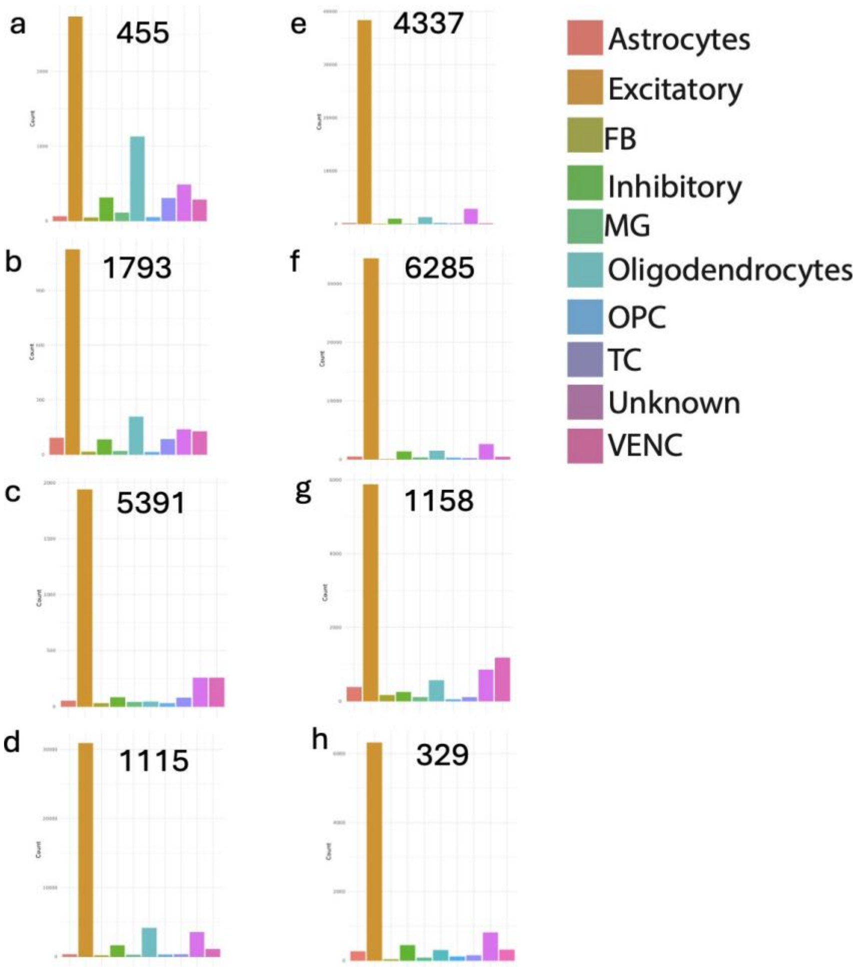

Figure S6 Cell Type Decomposition By Sample. **a-h)** Robust Cell Type Decomposition (RCTD) program results showing total number of singlet spots per cell type by sample, including astrocytes, excitatory neurons, fibroblasts (FB), inhibitory neurons, microglia (MG), oligodendrocytes, oligodendrocyte precursor cells (OPC), T cells (TC), unknown, and vascular endothelial cells (VENC). Young Adult age group are panels a,d,g, and h. Child age group are panels b,c, e and f. Color indicates cell type.

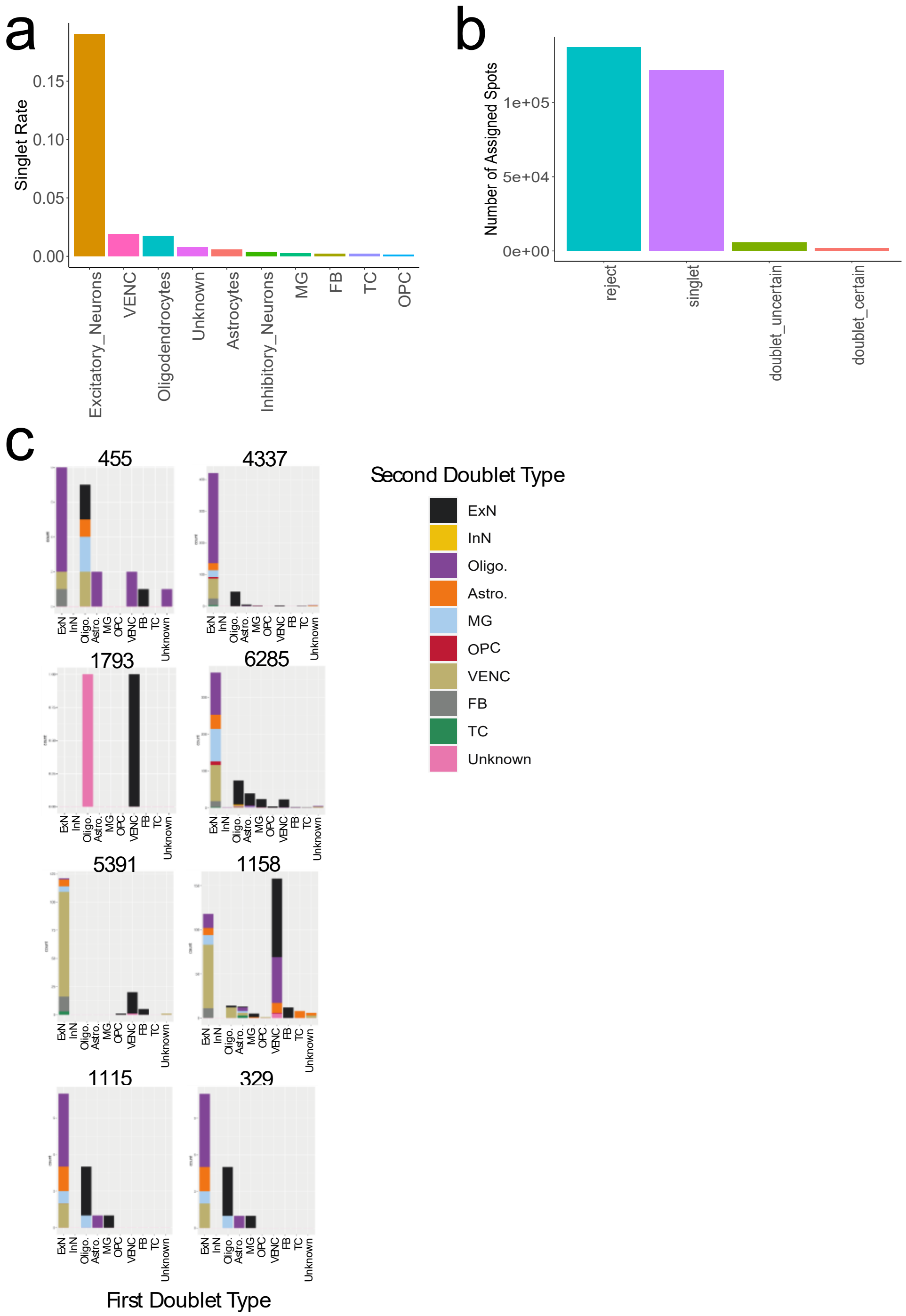

Figure S7 Cell Type Decomposition Overview. **a)** Number of assigned "singlets" from RCTD from all samples combined per cell type divided by the total number of detected barcodes in Illumina data (n=8 samples, n=482,777 barcodes). **b)** Number of assigned spot classes from all samples combined (n=8 samples) . **c)** Stacked bar plot by sample denoting the cell-type compositions of certain doublets. X axis lists the first identified cell type, and stacked color indicates the second cell identified type. Cell types include ExN=Excitatory Neurons, InN=Inhibitory Neurons, Oligo.= Oligodendrocytes, Astro.=Astrocytes, MG=Microglia, OPC=Oligodendrocyte Precursor Cells, VENC=Vascular Endothelial, FB=Fibroblasts, TC=T Cells, and Unknown.

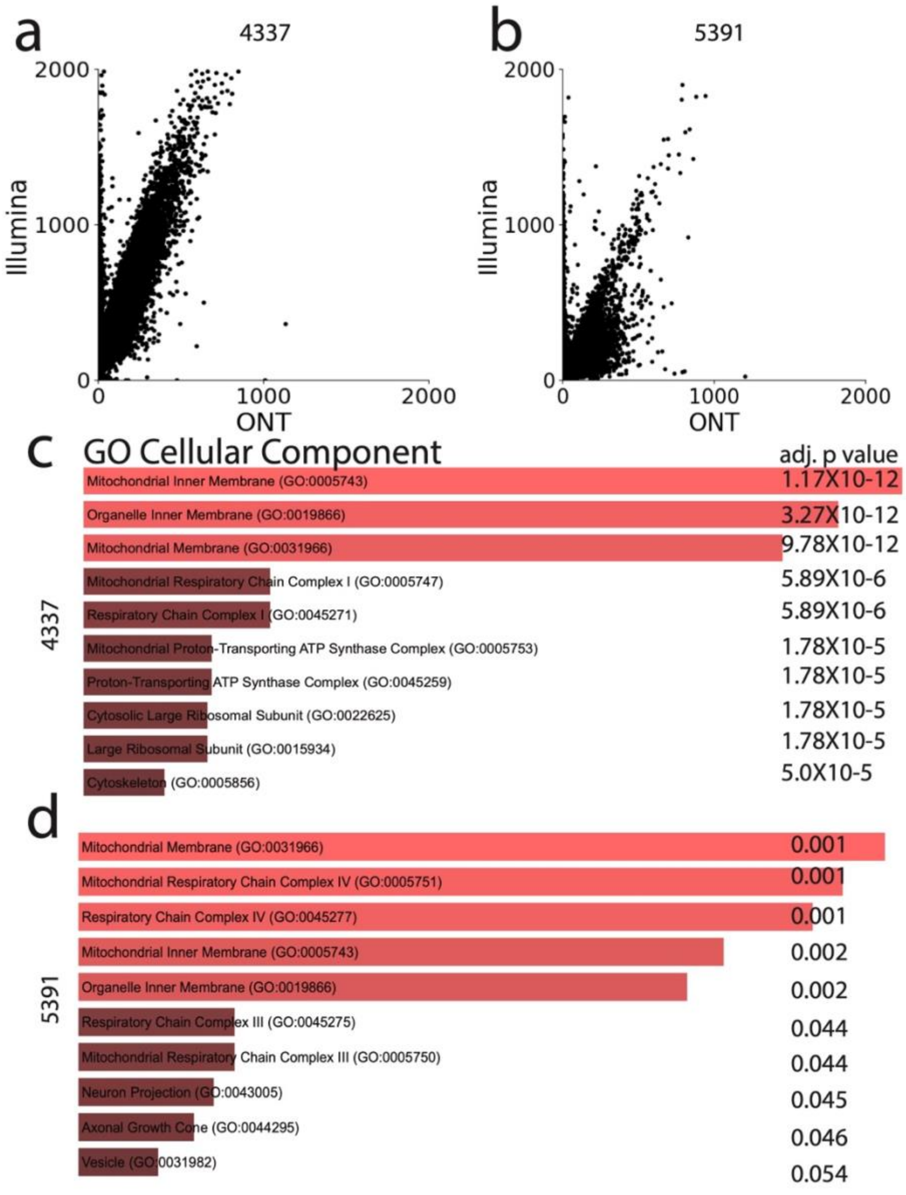

Figure S8 Short Read and Long Read Comparison. **a)** Dot plot of number of UMIs per barcode from a Long Exome dataset and Illumina dataset from the sample 4337. **b)** Dot plot of number of UMIs per barcode from a Long Exome ONT dataset and Illumina dataset from the sample 5391. **c)** EnrichR Gene Ontology analysis of the top 100 genes differentially regulated between barcodes which have high UMIs in Illumina data and low UMIs in ONT data for the sample 4337. **d)** EnrichR Gene Ontology analysis of the top 100 genes differentially regulated between barcodes which have high UMIs in Illumina data and low UMIs in ONT data for the sample 5391.

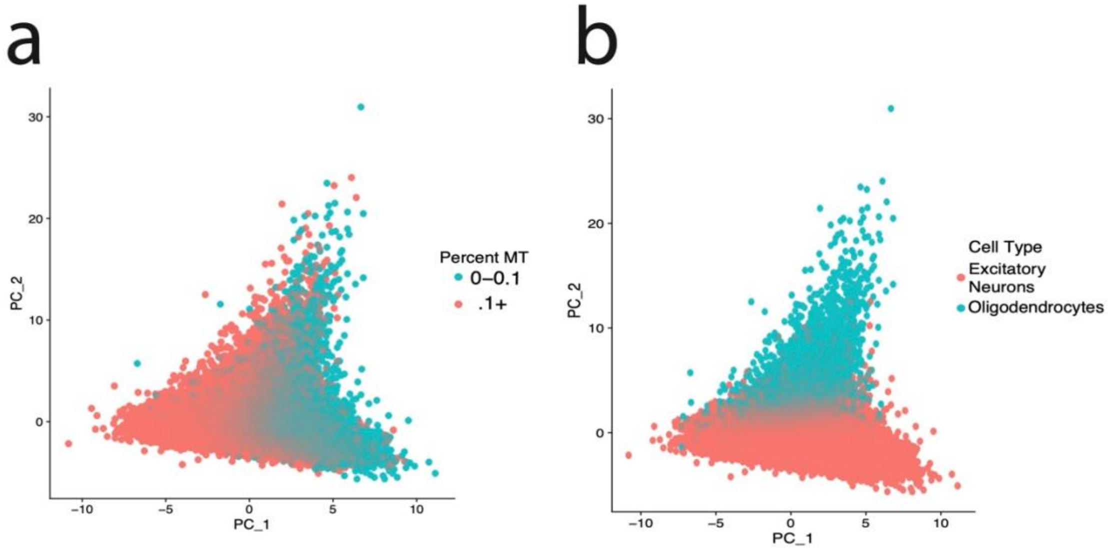

Figure S9 Principle Components. **a)** Spatial barcodes of singlets plotted by PC1 and PC2. Color indicates percent of reads coming from mitochondrial (MT) genes. Blue=Percent MT from 0-0.1; Red= Percent MT 0.1+. **b)** Spatial barcodes of singlets plotted by PC1 and PC2. Color indicates RCTD determined singlet cell type. Blue=Oligodendrocytes; Red=Excitatory Neurons.

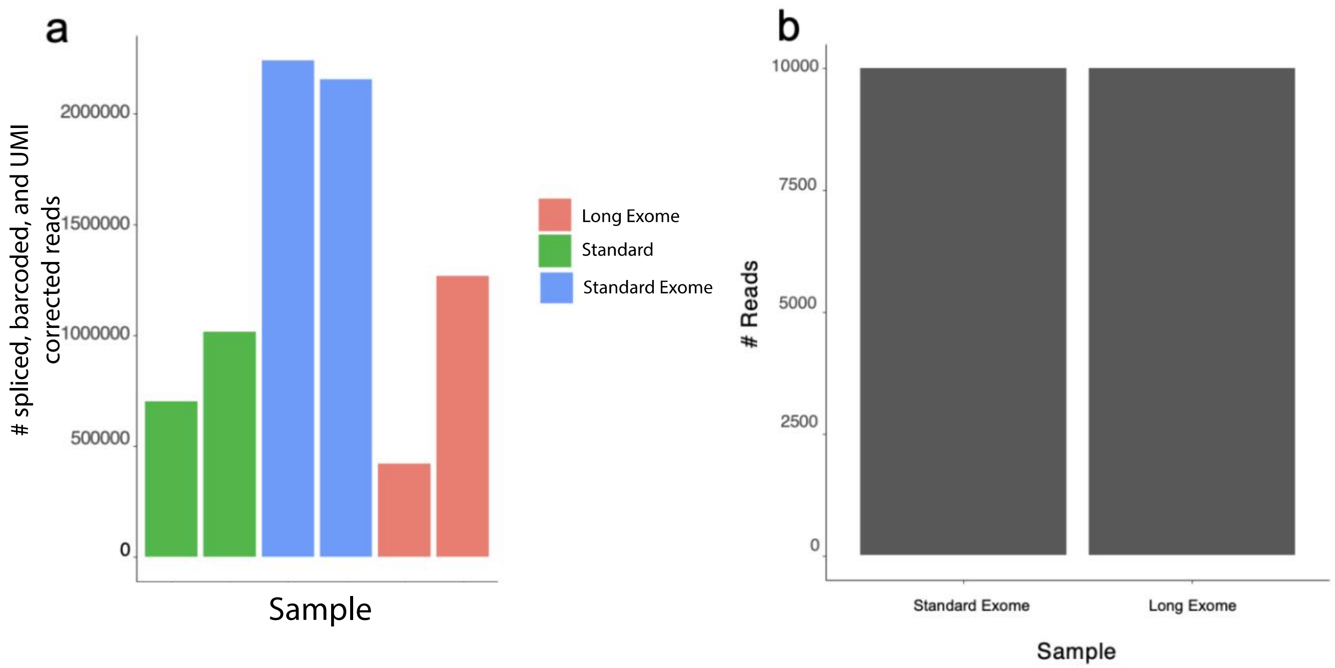

Figure S10 Number of Reads Per Sample Type. **a)** Number of reads from the 2 spatial samples shown in figure 2b and 2c. Color indicates data source. Standard: Untagmented cDNA acquired from the curio pipeline; Standard Exome: Untagmented cDNA acquired from the curio pipeline followed by an enrichment of molecules containing exons; Long Exome: Untagmented cDNA acquired from the curio pipeline followed by an enrichment of molecules containing exons and are optimally size selected. **b)** Number of reads from each single cell sample shown in figure 2d-f. Standard Exome: Untagmented cDNA acquired from 10X Genomics' single cell pipeline followed by an enrichment of molecules containing exons; Long Exome: Untagmented cDNA acquired from 10X Genomics' single cell pipeline followed by an enrichment of molecules containing exons and are optimally size selected.

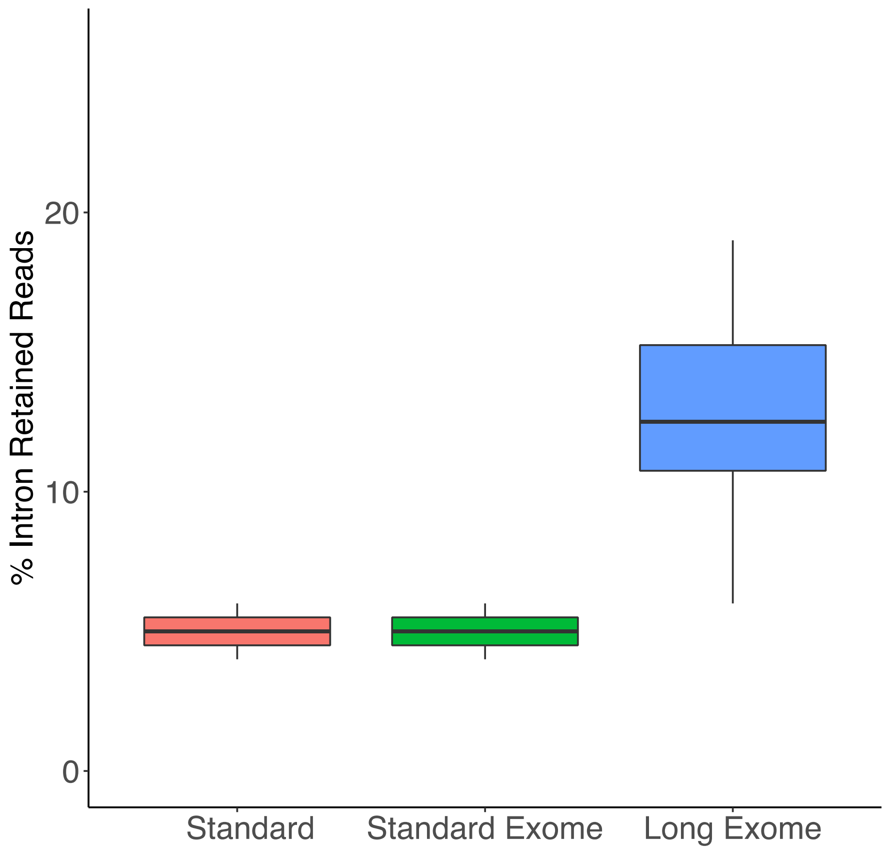

Figure S11 Intron Retention Per Dataset. Percent of all reads which contain retained introns across long read sequencing datasets. Center line indicates the median, upper bound at 75th percentile, lower bound at 25th percentile with whiskers at 1.5*interquartile range (IQR). Standard n=2, Standard Exome n=2, Long Exome n=8.

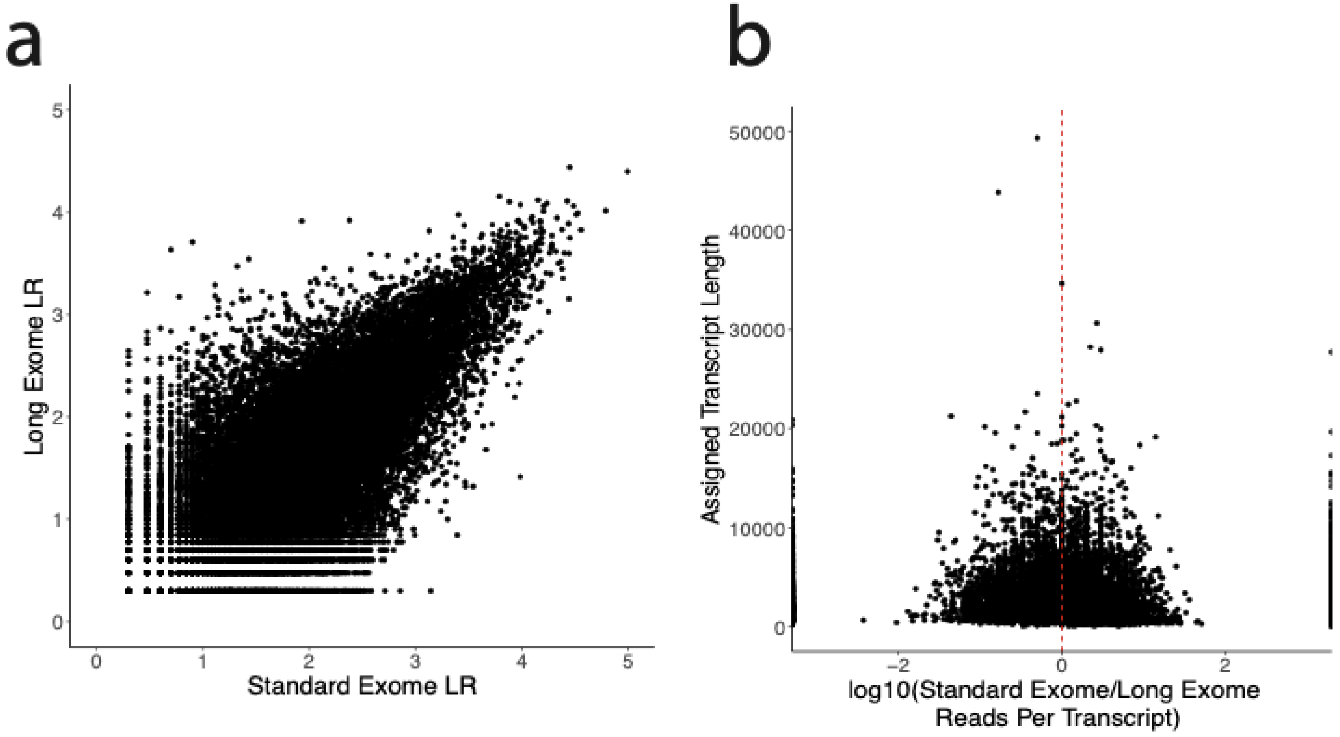

Figure S12 Standard Exome vs. Long Exome Comparison. **a)** Correlation of transcript expression between equally downsampled Standard Exome LR (n=2) and Long Exome LR datasets (n=2). **b)** Log10 of ratio reads per transcript (Standard Exome LR/Long Exome LR) by assigned transcript length. Red line indicates a value of 0.

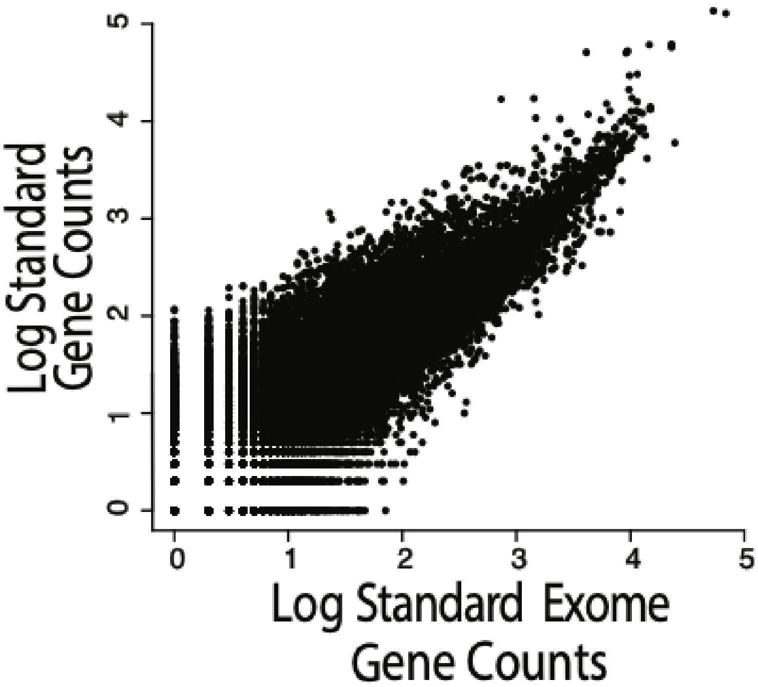

Figure S13. Log10 of the number of reads per gene between Standard Exome and Control No Exome datasets.

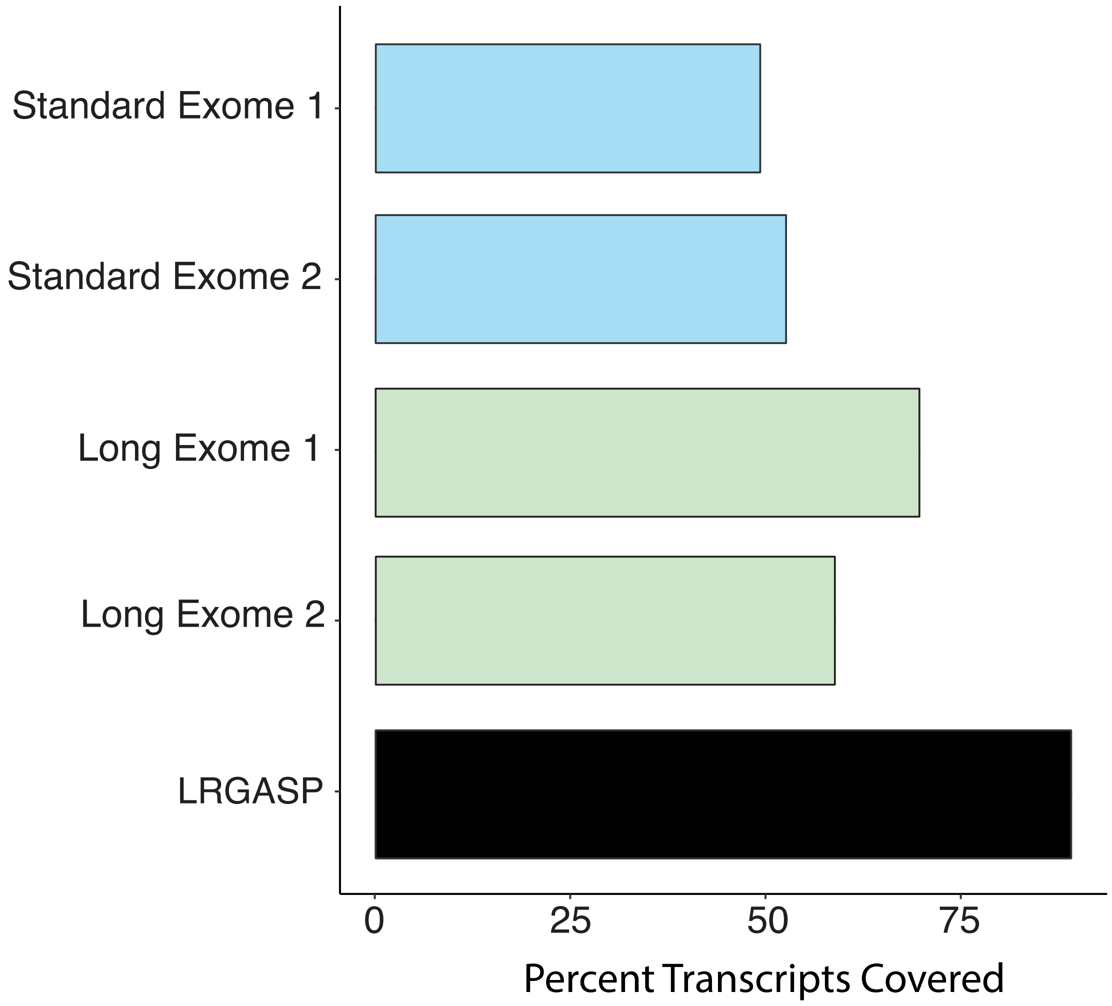

Figure S14. Percent of the transcript per base which is spanned by reads in each dataset. Dataset 1=455; Dataset 2=5391; LRGASP data is bulk, full length RNA seq data from the LRGASP consortium.

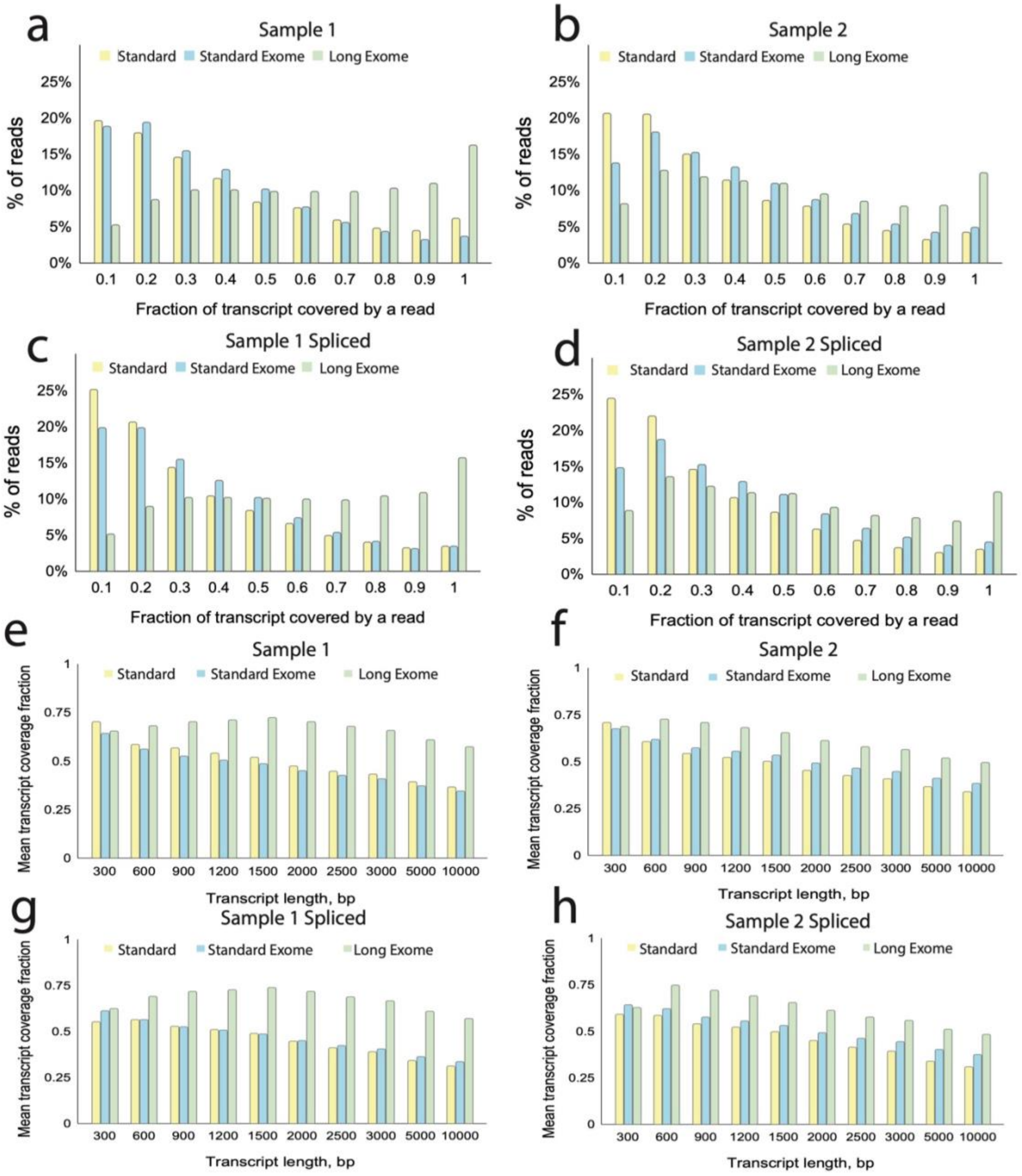

Figure S15 Fraction of read covered statistics. **a)** % of reads which fall into bins of fraction of transcript covered in sample 1 across standard, standard exome, and long exome datasets. **b)** Same as (a) but for sample 2 across standard, standard exome, and long exome datasets. **c)** % of spliced reads which fall into bins of fraction of transcript covered in sample 1 across standard, standard exome, and long exome datasets. **d)** Same as (c) but for sample 2 across standard, standard exome, and long exome datasets. **e)** Average fraction of transcript covered binned by reference transcript length covered in sample 1 across standard, standard exome, and long exome datasets. **f)** Same as (e) but for sample 2 across standard, standard exome, and long exome datasets. **g)** Fraction of average spliced transcript covered binned by transcript length covered in sample 1 across standard, standard exome, and long exome datasets**. h)** Same as (g) but for sample 2 across standard, standard exome, and long exome datasets. Sample 1 indicates sample 455 and sample 2 indicates sample 5391. Yellow indicates data from Standard, blue from Standard Exome, and Green from Long Exome.

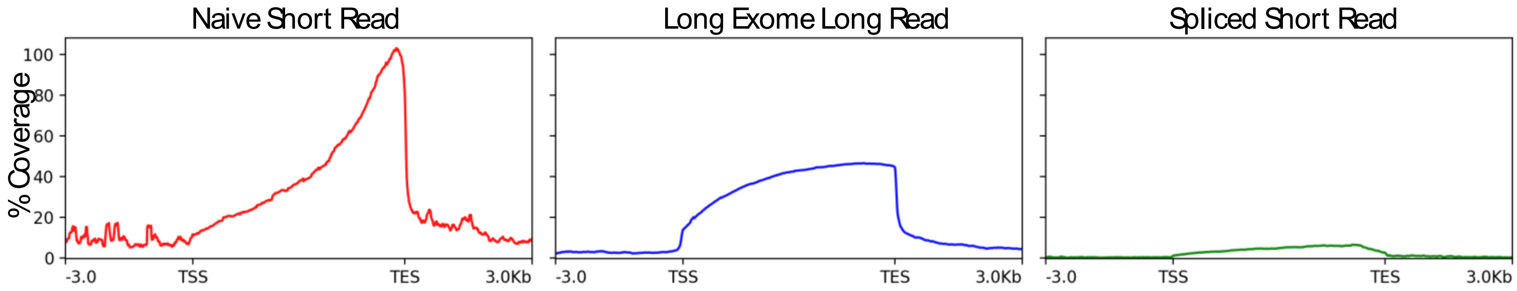

Figure S16. Metagene plot showing exonic coverage of annotated genes with normalized length. Red shows coverage from the Naïve Short Read data, blue trace shows coverage from the Long Exome LR data, and green trace shows coverage from the Spliced Short read data. TSS indicates transcription start site and TES indicates transcription end site.

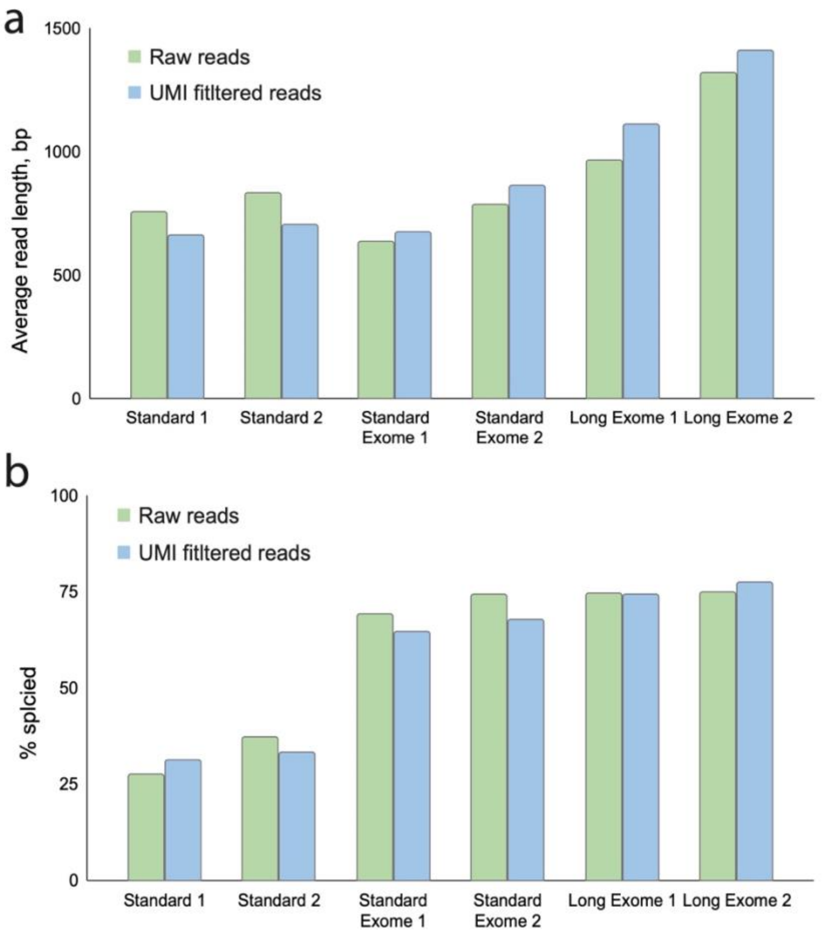

Figure S17 UMI Filtering. **a)** Average read length per sample separated by all reads (raw) and UMI filtered. **b)** % of spliced reads per sample separated by all reads (raw) and UMI filtered.

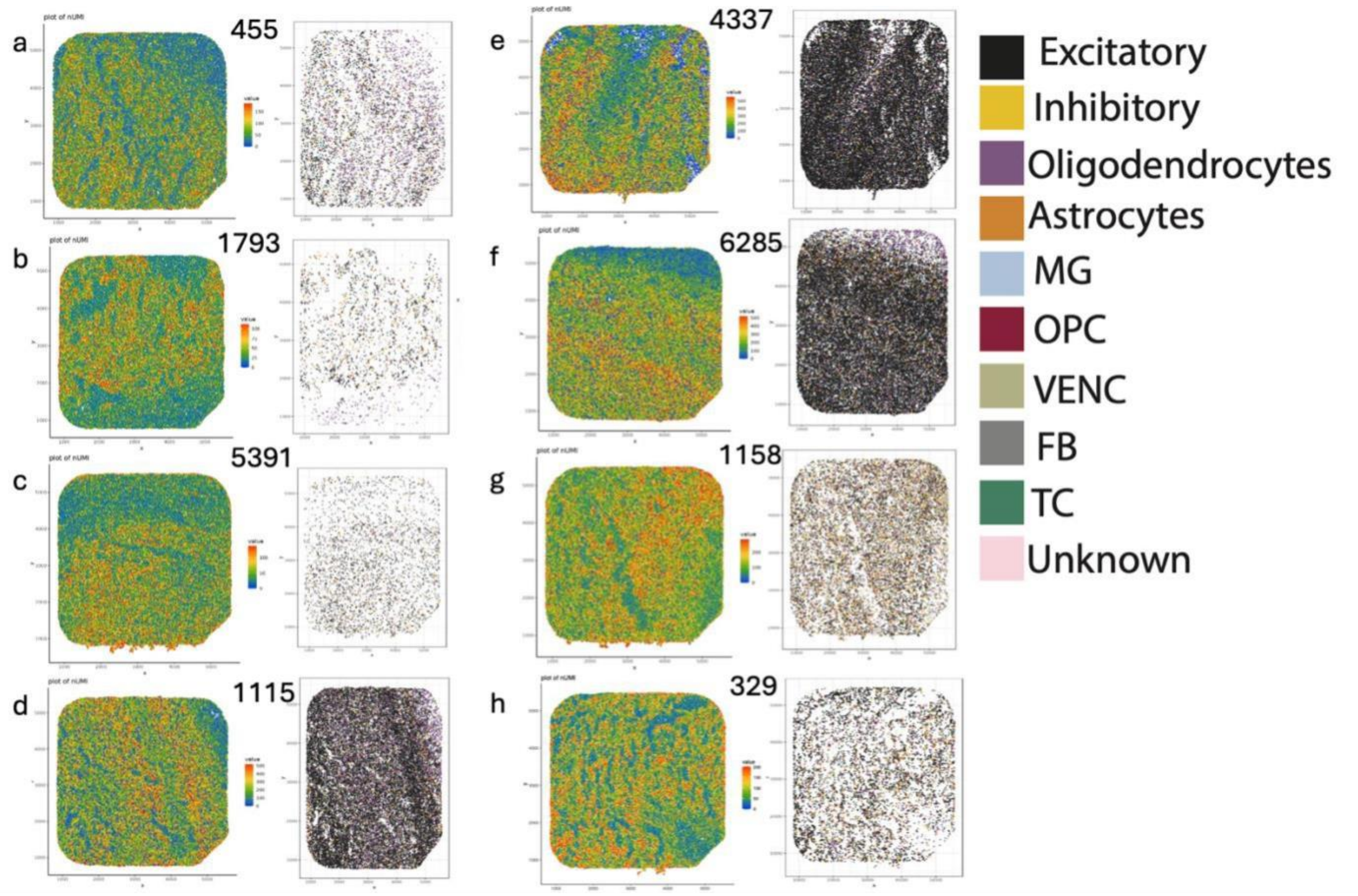

Figure S18 UMIs and Cell types Per Slide. **a-h)** Left panel: Number of UMIs per spot plotted by spatial location for each sample. Right panel: RCTD predicted singlets plotted by cell type and spatial location for each sample, including astrocytes, excitatory neurons, fibroblasts (FB), inhibitory neurons, microglia (MG), oligodendrocytes, oligodendrocyte precursor cells (OPC), T cells (TC), unknown, and vascular endothelial cells (VENC).

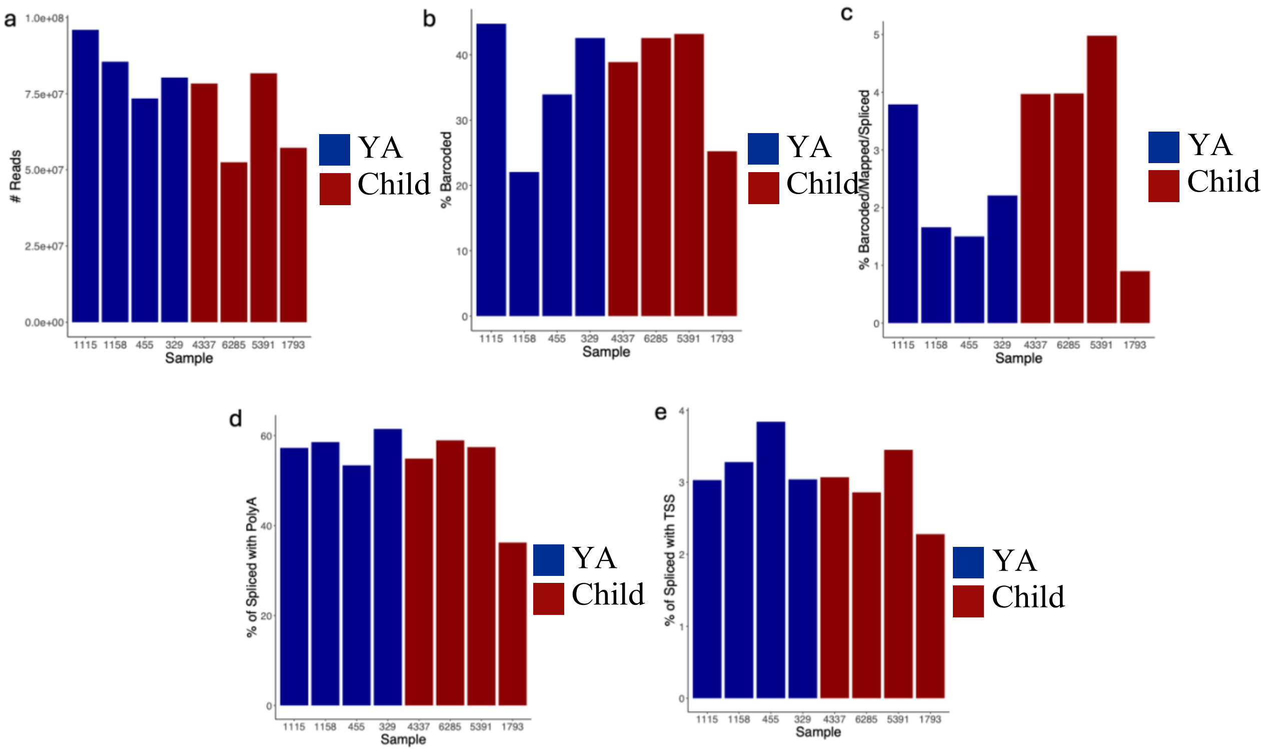

Figure S19 Long Read Statistics. **a)** Number of Oxford Nanopore Technology (ONT) reads sequenced per sample. **b)** Percent of ONT reads containing proper barcode with a barcode score of at least 13 per sample. **c)** Percent of ONT reads which are barcoded (score >=13), mapped to hg38, and spliced as defined by Spl-isoquant. **d)** Percent of the barcoded, mapped, and spliced reads with a defined PolyA site. **e)** Percent of the barcoded, mapped, and spliced reads with a defined TSS site.

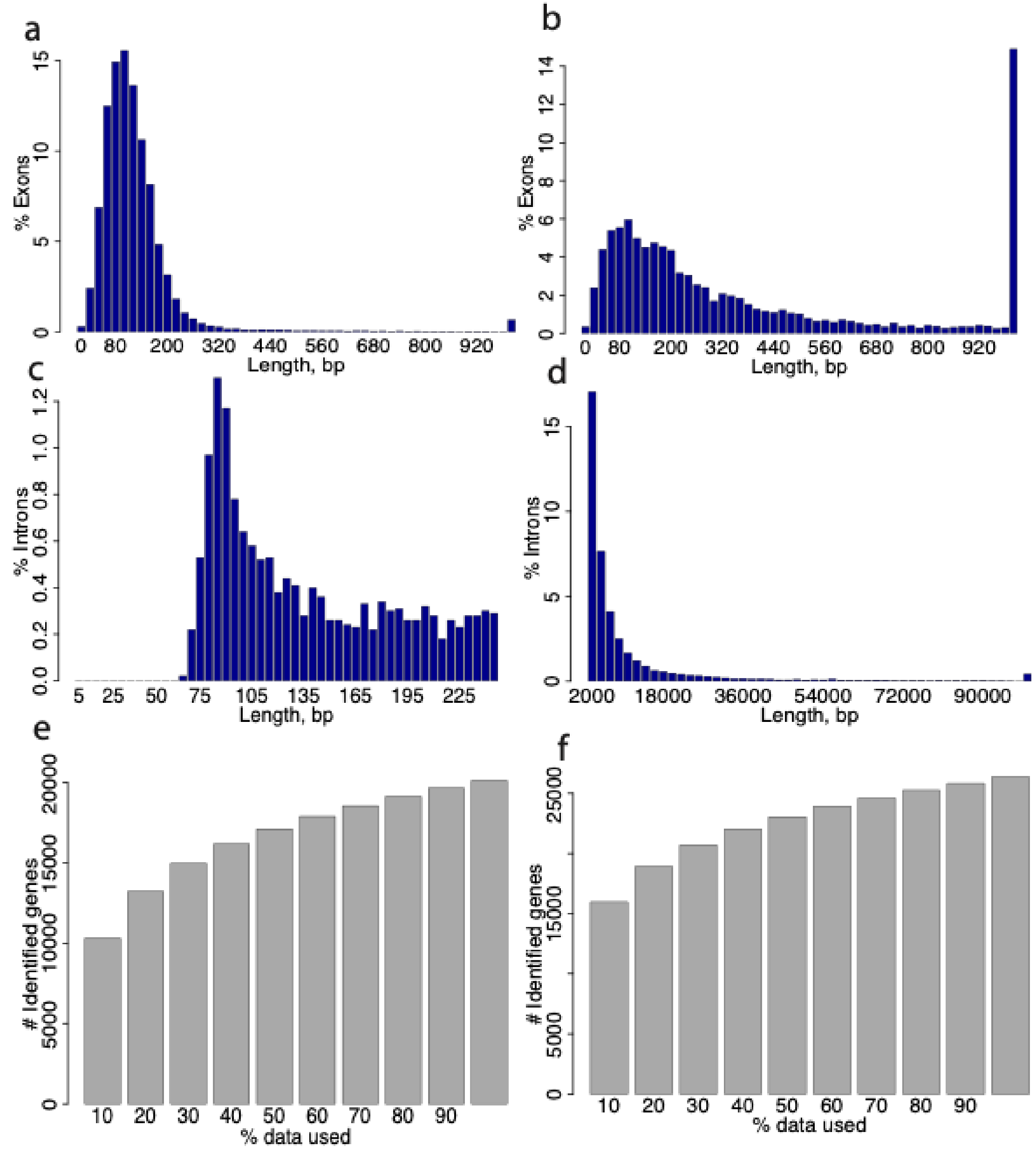

Figure S20 Exon Length and Number of Genes. **a)** Length distribution of internal exons where last bar corresponds to all exons >= 1000 bp. **b)** Length distribution of terminal exons where last bar corresponds to all exons >= 1000 bp. **c)** Intron length distribution between sizes 0-250 bp. **d)** Intron length distribution where last bar corresponds to all exons >= 100k bp. **e)** Number of identified genes with >=10 UMIs when sampling decreasing percentages of spliced data. **f)** Number of identified genes with >=3 UMIs when sampling decreasing percentages of spliced data.

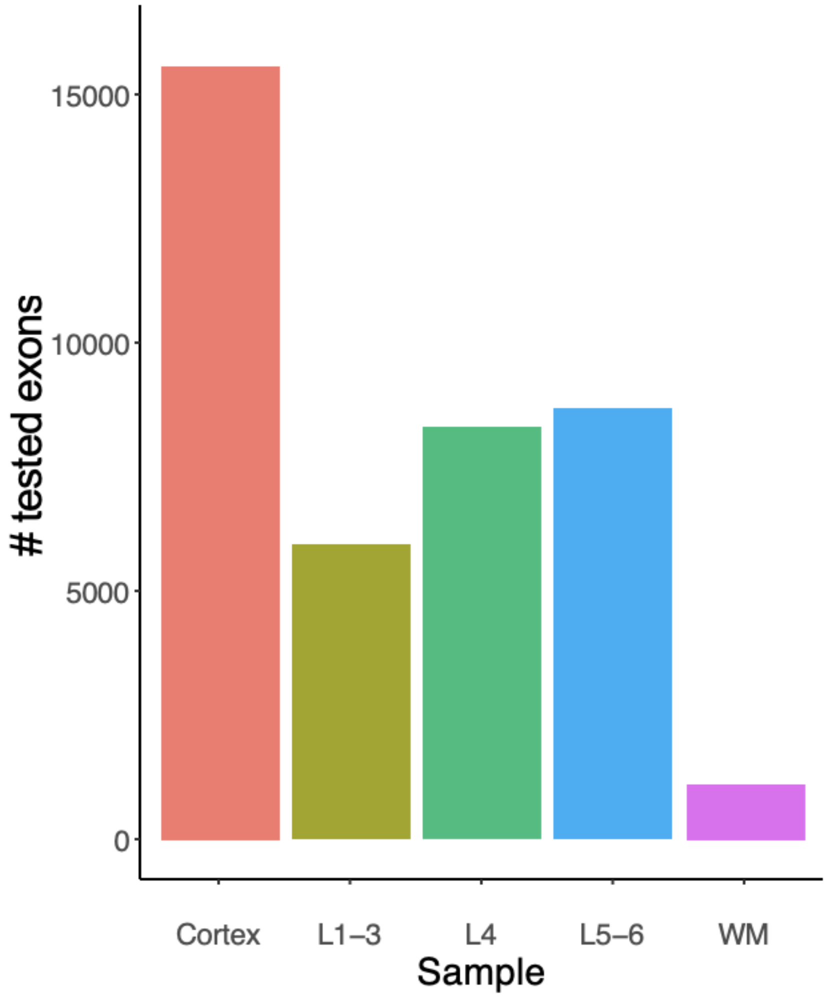

Figure S21. Number of tested exons for each area-specific comparison by age. Areas include Cortex, Layers 1-3 (L1-3), Layer 4 (L4), Layers 5-6 (L5-6), and white matter (WM).

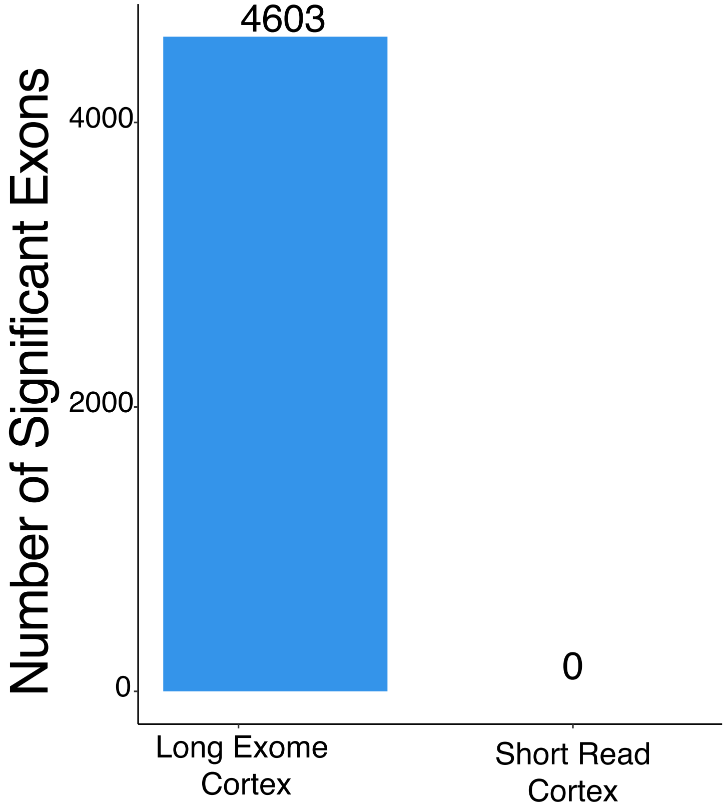

Figure S22. Number of significant exons identified to be regulated across age in the cortex by dataset. Long Exome Cortex: ONT data which is exome enriched and long-molecule selected. Short Read: Spliced short reads from the Naïve short read dataset.

a

Number of Reads Per Condition (Child vs. Young Adult)	|dPSI|					
	0	**0.1**	**.2**	**.3**	**.4**	**.5**
50-249	0	18	63	87	98	100
40-49	0	0	12	65	90	99
30-39	0	0	1	34	79	90
20-29	0	0	1	14	43	78
10-19	0	0	0	3	14	38
0-9	0	0	0	0	0	0

b

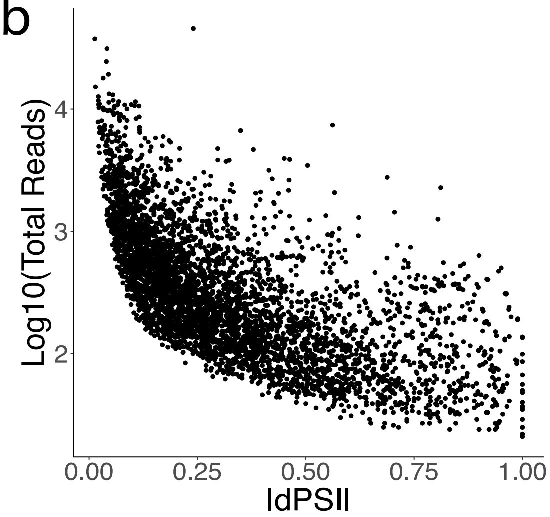

Figure S23 Downsampling experiments to determine minimum read numbers per exon. **a)** Matrices with 1000 reads per condition with predefined |dPSI| were downsampled to read counts of [0,9], [10,19], [20,29], [30,39], [40,49], [50,249] per condition. Values indicate the fraction of matrices that pass Benjamini-Yekutieli correction for multiple testing per |deltaPSI| and read number combination. **b)** Significant exons from the Cortex Child vs. Young Adult comparison plotted by |dPSI| and Log10(Total Reads).

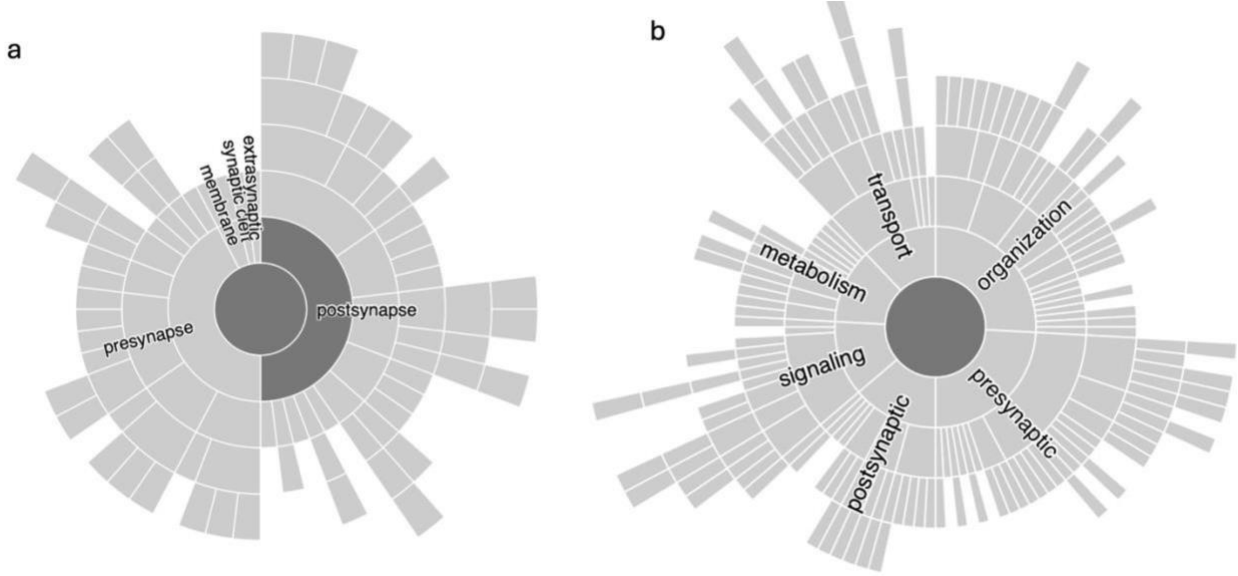

Figure S24 SynGo results for WM. **a)** SynGo location enrichment analysis of genes which are differentially spliced in white matter across age. **b)** SynGo function enrichment analysis of genes which are differentially spliced in white matter across age.

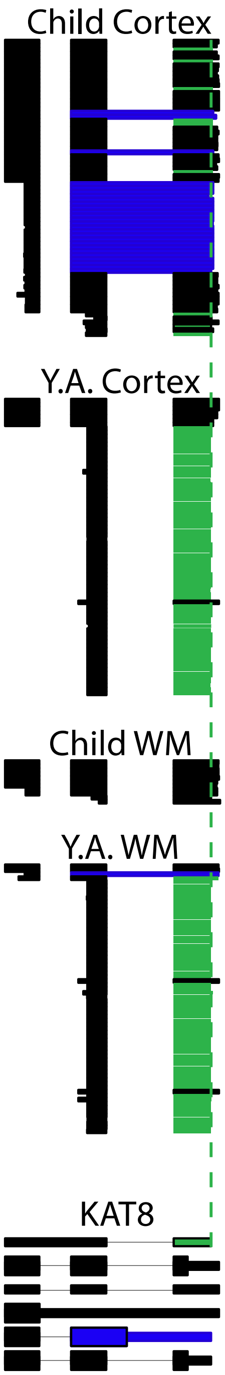

Figure S25. ScisorWiz plot of the gene KAT8. Top row: spliced, barcoded, and UMI corrected reads from the child age group which are located in the cortex. 2nd Row: spliced, barcoded, and UMI corrected reads from the young adult age group which are located in the cortex. 3rd Row: spliced, barcoded, and UMI corrected reads from the child age group which are located in white matter. Last Row: spliced, barcoded, and UMI corrected reads from the young adult age group which are located in white matter. Color indicates alternative last exon usage. Green: chr16:31,131,195-31,131,359; Blue: chr16:31,130,746-31,131,358. Black: All others. Green dotted line: aligns with start of exon chr16:31,131,195-31,131,359 (green label).

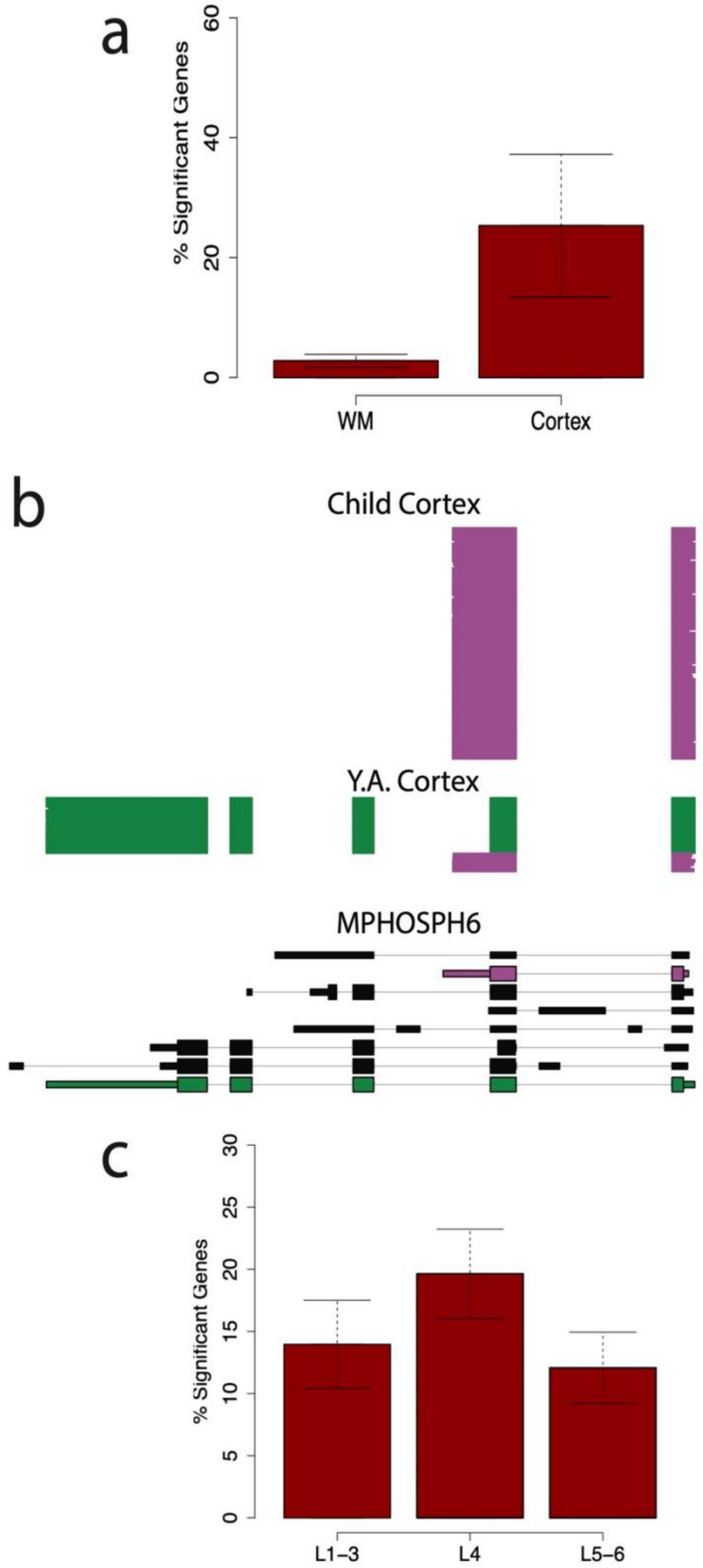

Figure S26 Full Length Differential Isoforms. **a)** Percent significant genes which exhibit isoform changes across age, separated by brain region; WM: White Matter. **b)** ScisorWiz plot of the gene MPHOSPH6. Isoform1 is labeled in pink and Isoform2 is labeled in green. **c)** Percent significant genes which exhibit isoform changes separated by layer. Error bars in a,c indicate 95% confidence intervals. Values in a,c are from 4 samples combined per age group.

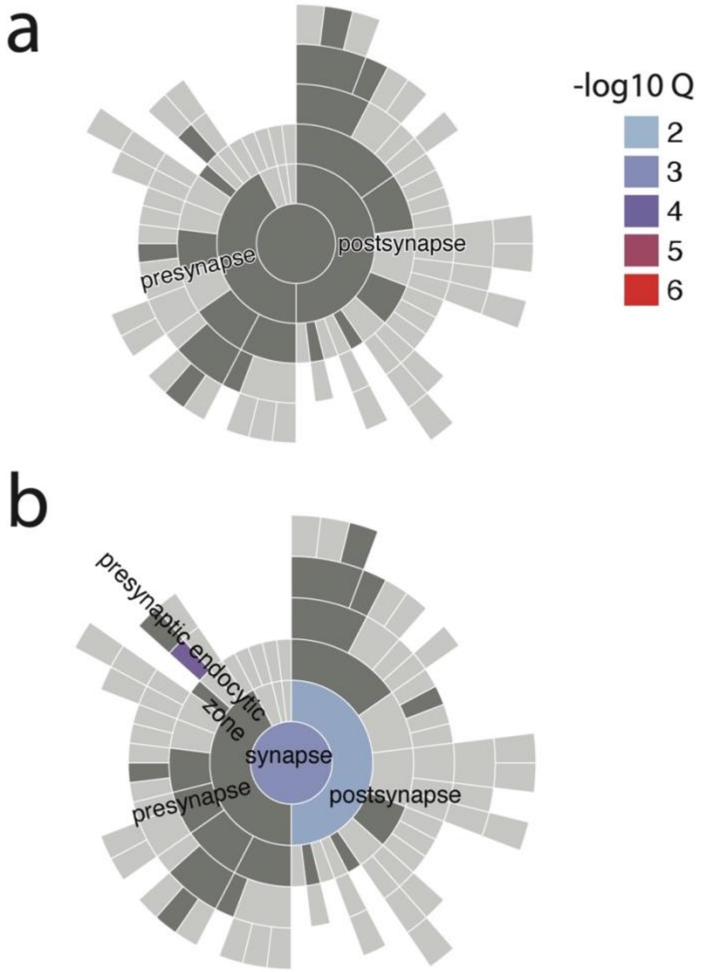

Figure S27 SynGo Enrichment Per Layer. a) SynGo enrichment plots of genes with significant alternative exons in layers 1-3 across age. b) SynGo enrichment plots of genes with significant alternative exons in layers 5-6 across age.

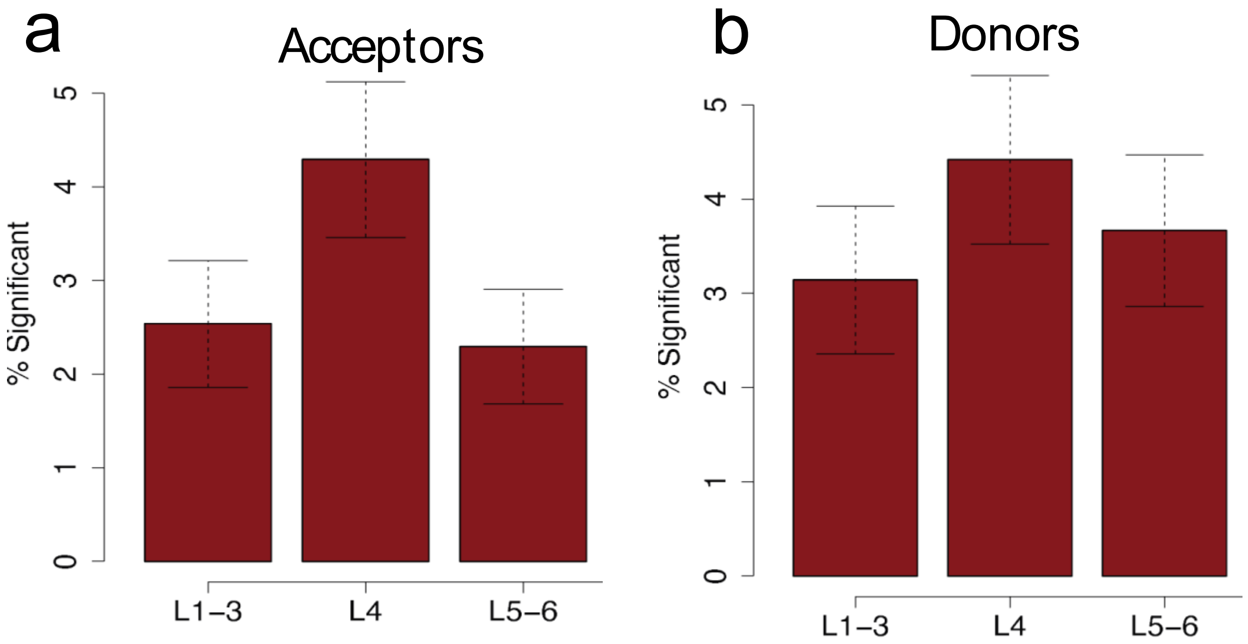

Figure S28 Alternative Acceptors and Donors. **a)** Percent significant alternative acceptor sites by layer from 8 samples combined. **b)** Percent significant alternative donor sites by layer from 4 samples combined per age group. Error bars indicate 95% confidence intervals.

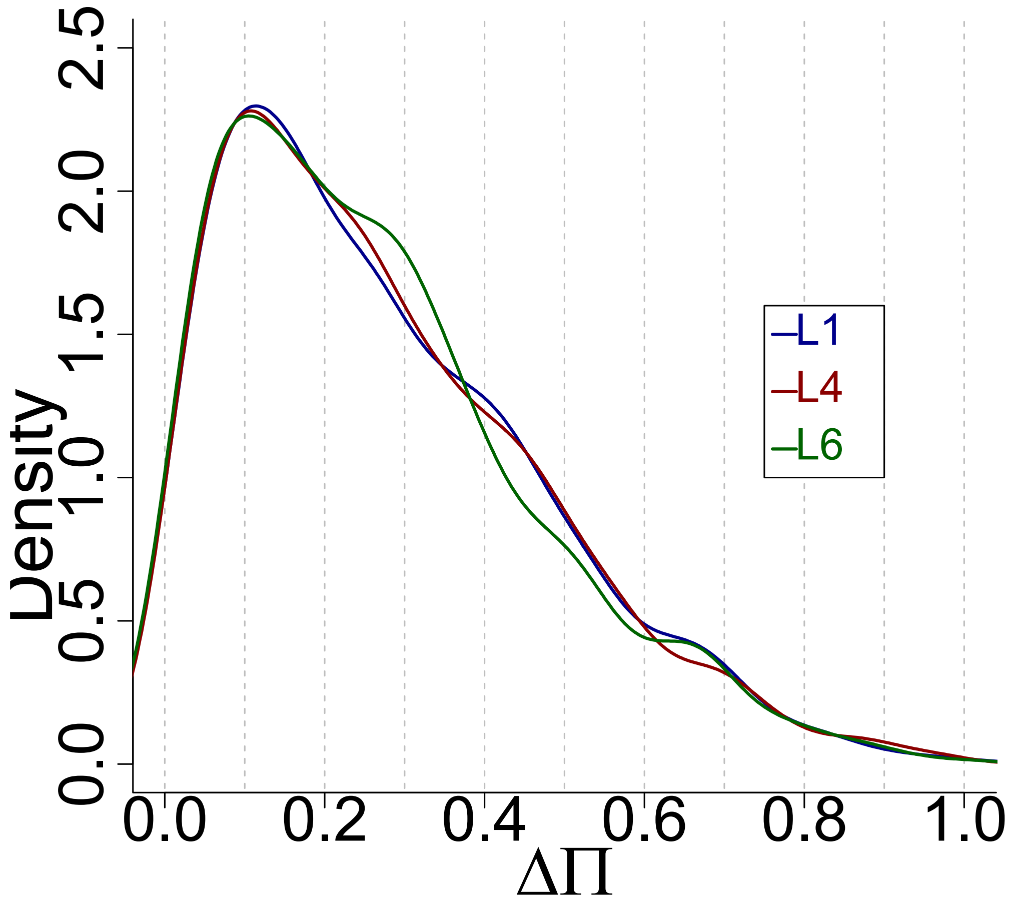

Figure S29. Density of |deltaPI| values identified for significant alternative Poly(A)-site usage. Lines are drawn by color to indicate layer; Blue = L1-3, Red=L4, Green=L5-6.

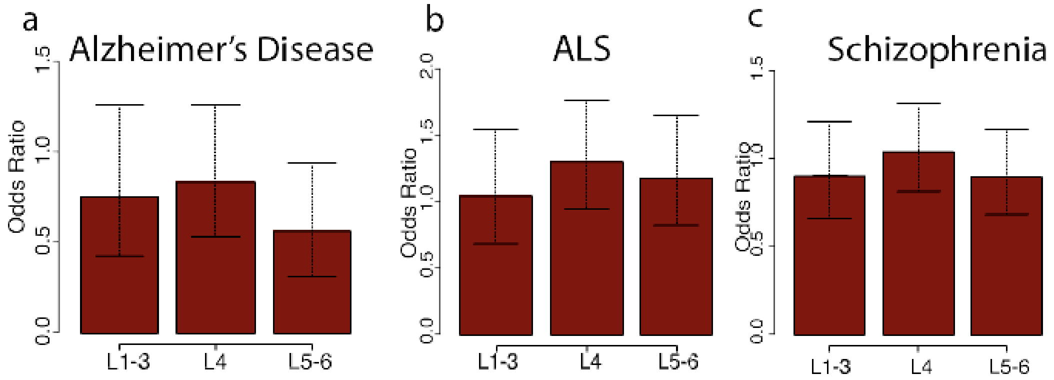

Figure S30 Disease Associated Genes Per Layer. **a)** Odds ratio comparing significant group (|dPSI| > .5 and FDR < .05) v background group (|dPSI| < .1 and FDR > .05) of Alzheimer's Diseases associated genes. **b)** Odds ratio comparing significant group v background group of ALS associated genes. **c)** Odds ratio comparing significant v background group of Schizophrenia associated genes. Error bars indicate 95% confidence intervals. N=8

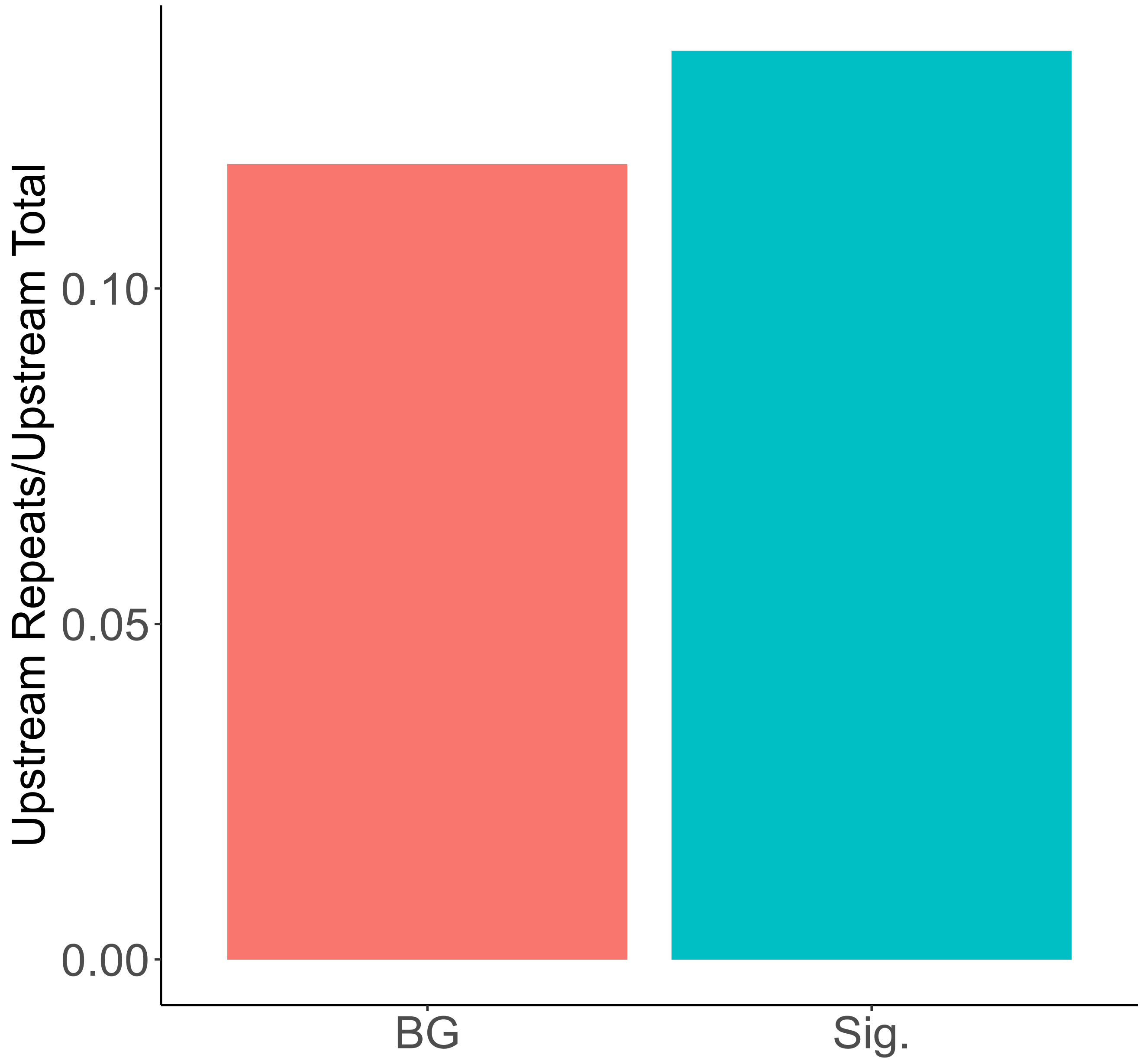

Figure S31. Ratio of sequences with repetitive elements found to total number of sequences per group. Sig: Sequences found upstream of exons with |dPSI| > 0.5 and FDR < .05. BG: Sequences found upstream of exons with |dPSI| < 0.05 and FDR > .05. 2-sided Fisher test, FDR = 0.334.

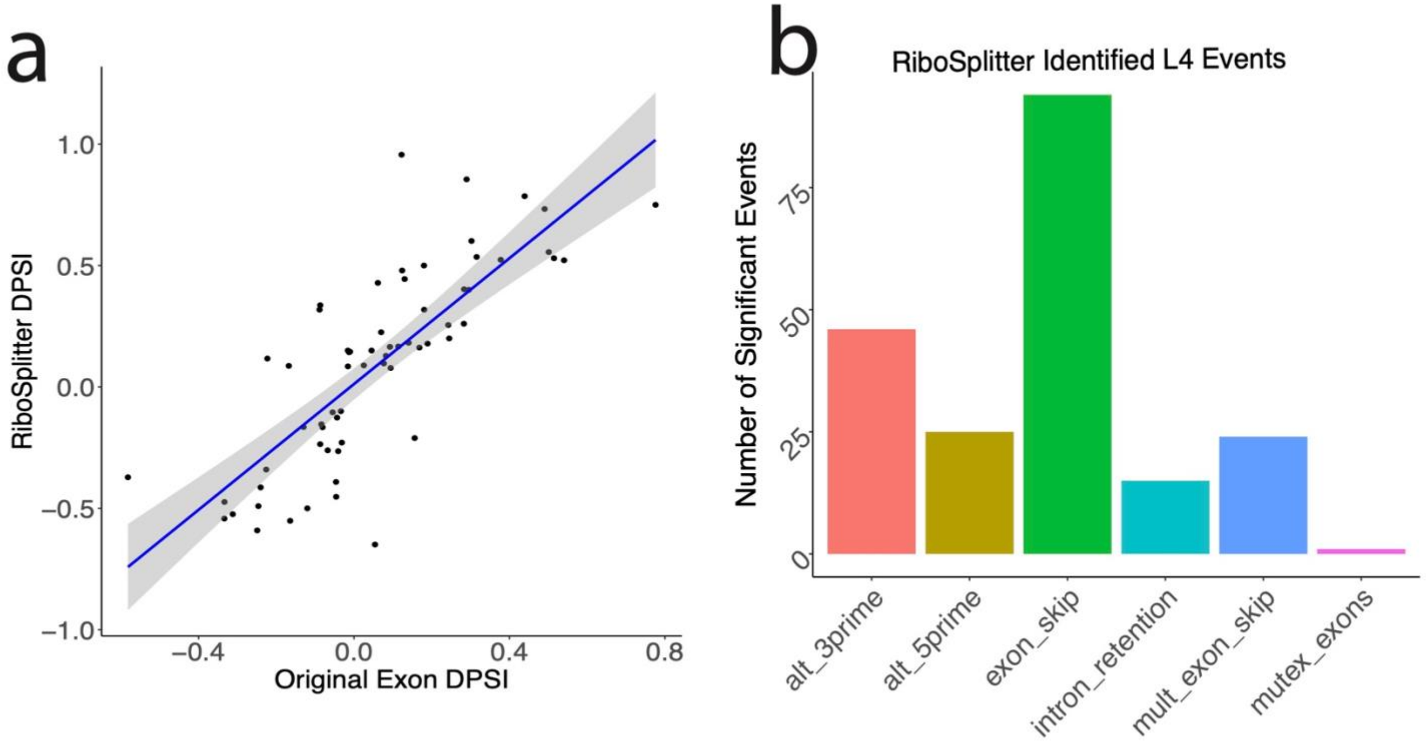

Figure S32 RiboSplitter Results. **a)** Correlation of the DPSIs of exons in L4 calculated as in Figure 6(a) compared to DPSIs calculated by RiboSplitter. Blue line represents the line of best fit. **b)** Number of significant events per category found by RiboSplitter.
