# Supplementary material for: A spatial long-read approach at near-single-cell resolution reveals developmental regulation of splicing and polyadenylation sites in distinct cortical layers and cell types
Source: Nat Commun. 2025 Aug 29;16:8093. doi: 10.1038/s41467-025-63301-9 (PMC12397408; doi:10.1038/s41467-025-63301-9)
Supplement: Supplementary file 2 — Description of Additional Supplementary Files [file 41467_2025_63301_MOESM2_ESM.docx]

Description of Additional Supplementary Files

Supplementary Data 1: Subject details for human visual cortex samples.

Supplementary Data 2: A simulation of truncation from ONT reads and length and assignment recall and precision.

Supplementary Data 3: A breakdown from 2 samples across methods (Standard, Standard Exome, and Long Exome) of read filtering and statistics per barcode and gene from the original set of reads.

Supplementary Data 4: Overview of Long Exome reads per gene, transcript, and exon per sample. Y.A. indicates Young Adult age group.

Supplementary Data 5: Total number of tested and significant exons, Poly(A) genes, and isoforms between age groups.

Supplementary Data 6: Protein domains and genes affected by splicing events in layer 4.
