# Supplementary material for: A spatial long-read approach at near-single-cell resolution reveals developmental regulation of splicing and polyadenylation sites in distinct cortical layers and cell types
Source: Nat Commun. 2025 Aug 29;16:8093. doi: 10.1038/s41467-025-63301-9 (PMC12397408; doi:10.1038/s41467-025-63301-9)
Supplement: Supplementary file 9 — Reporting Summary [file 41467_2025_63301_MOESM9_ESM.pdf]

nature portfolio

Corresponding author(s): Tilgner, Hagen U.

Last updated by author(s): 7/28/2025

# Reporting Summary

Nature Portfolio wishes to improve the reproducibility of the work that we publish. This form provides structure for consistency and transparency in reporting. For further information on Nature Portfolio policies, see our Editorial Policies and the Editorial Policy Checklist.

## Statistics

For all statistical analyses, confirm that the following items are present in the figure legend, table legend, main text, or Methods section.

| n/a | Confirmed | |
|---|---|---|
| ☐ | ☒ | The exact sample size (*n*) for each experimental group/condition, given as a discrete number and unit of measurement |
| ☐ | ☒ | A statement on whether measurements were taken from distinct samples or whether the same sample was measured repeatedly |
| ☐ | ☒ | The statistical test(s) used AND whether they are one- or two-sided<br>*Only common tests should be described solely by name; describe more complex techniques in the Methods section.* |
| ☐ | ☒ | A description of all covariates tested |
| ☐ | ☒ | A description of any assumptions or corrections, such as tests of normality and adjustment for multiple comparisons |
| ☐ | ☒ | A full description of the statistical parameters including central tendency (e.g. means) or other basic estimates (e.g. regression coefficient) AND variation (e.g. standard deviation) or associated estimates of uncertainty (e.g. confidence intervals) |
| ☐ | ☒ | For null hypothesis testing, the test statistic (e.g. *F*, *t*, *r*) with confidence intervals, effect sizes, degrees of freedom and *P* value noted<br>*Give P values as exact values whenever suitable.* |
| ☒ | ☐ | For Bayesian analysis, information on the choice of priors and Markov chain Monte Carlo settings |
| ☒ | ☐ | For hierarchical and complex designs, identification of the appropriate level for tests and full reporting of outcomes |
| ☐ | ☒ | Estimates of effect sizes (e.g. Cohen's *d*, Pearson's *r*), indicating how they were calculated |

*Our web collection on statistics for biologists contains articles on many of the points above.*

## Software and code

Policy information about availability of computer code

| | |
|---|---|
| Data collection | Code for data collection was used in downsampling experiments. In downsampling experiments, custom code was used to sample equal reads from each individual, followed by sampling a subset of the number of reads  and the number of genes. We then calculated the DPSI and % significant exons, and then repeated this cycle 100x. In several figures we plot the distribution of % significant exons across 100 cycles. TransNanosim (https://github.com/andrewprzh/lrgasp-simulation) was used to simulate ONT data. |
| Data analysis | To analyze long read data, we use Spl-ISO-quant, a custom made software which can be found at: https://github.com/algbio/spl-IsoQuant. To analyze short read data, we used the Curio Seeker Runner 2.0.2, which is included when purchasing Curio slides. We additionally used the RCTD package to identify cell types, which can be found here: https://github.com/dmcable/spacexr. We also used the FindMarkers function from Seurat 5.1.0 paired with clusterprofiler 4.2.2 for gene expression analysis. Protein domain modelling was performed with SUPERFAMILY, InterProScan, and AlphaFold3. Protein Domain analysis was performed with Spladder and Ribosplitter. |

For manuscripts utilizing custom algorithms or software that are central to the research but not yet described in published literature, software must be made available to editors and reviewers. We strongly encourage code deposition in a community repository (e.g. GitHub). See the Nature Portfolio guidelines for submitting code & software for further information.

# Data

Policy information about availability of data

All manuscripts must include a data availability statement. This statement should provide the following information, where applicable:

- Accession codes, unique identifiers, or web links for publicly available datasets
- A description of any restrictions on data availability
- For clinical datasets or third party data, please ensure that the statement adheres to our policy

All data used for this study are uploaded to the National Institute of Health's Sequence Reads Archive (SRA) under submission PRJNA1116561.

# Research involving human participants, their data, or biological material

Policy information about studies with human participants or human data. See also policy information about sex, gender (identity/presentation), and sexual orientation and race, ethnicity and racism.

| | |
|---|---|
| Reporting on sex and gender | Fresh frozen human brain samples from the Visual cortex (from brodmann area 17) were obtained from the NIH Neurobiobank. We received 4 male samples aged 8-11 years old and 4 male samples aged 16-19 years old. We requested these age groups as our study is investigating spatially regulated isoforms across age, specifically during the time of puberty. We chose to do this study with all male samples as females are known to undergo earlier time frames of puberty, as well as different time periods of hormonal spikes. In this scenario, it would pose a challenge to differentiate spatially regulated RNA isoforms which may be regulated by the estrous cycle and/or sex-based hormones. |
| Reporting on race, ethnicity, or other socially relevant groupings | Of the 4 8-11 year old samples, the race of 2 samples was "black", 1 sample was "white", and 1 sample was "unknown". Of the 4 16-19 year old samples, the race of 3 samples was "white" and the race of 1 sample was "black". We attempted to match the groups proportions as much as possible given the NIH NeuroBioBank's sample availability. We determined that there were no substantial race-based gene expression differences by combining and clustering gene expression of samples together using the Seurat pipeline. |
| Population characteristics | Of the 4 8-11 year old samples, 2 were aged 8 and 2 were aged 11. Of the 4 16-19 year old samples, 2 were aged 16, 1 was aged 18, and 1 was aged 19. We attempted to age-match the groups such that the average age of samples from each group was close to the average value of each age group (eg. 8-11, median = 9.5; 16-19, median=17.5), given the NIH NeuroBioBank's sample availability. |
| Recruitment | Human tissues were acquired from the NIH NBB. Human research participants were not involved. |
| Ethics oversight | an MTA was approved by both NIH NBB and Weill Cornell. |

Note that full information on the approval of the study protocol must also be provided in the manuscript.

# Field-specific reporting

Please select the one below that is the best fit for your research. If you are not sure, read the appropriate sections before making your selection.

☒ Life sciences ☐ Behavioural & social sciences ☐ Ecological, evolutionary & environmental sciences

For a reference copy of the document with all sections, see nature.com/documents/nr-reporting-summary-flat.pdf

# Life sciences study design

All studies must disclose on these points even when the disclosure is negative.

| | |
|---|---|
| Sample size | No statistical methods were used to determine sample size. We chose 4 samples in each group (child = 8-11 y.o and young adult = 16-19 y.o.) due the combined variables of fresh frozen, non-pulvarized, and high RNA integrity of human tissue availability. Although our data suggests examples of individual variation, our downsampling method ensures we can identify many cases which are not due to individual variation. |
| Data exclusions | No data was excluded. |
| Replication | Experiments to replicate our findings were not included in this manuscript. |
| Randomization | Experimental groups were attempted to be matched by age-group median and race proportions to the best of our ability given the NIH NeuroBioBank's sample availability. |
| Blinding | Blinding was not possible during this study as the request for tissues was performed by the same scientist who performed experiments and data analysis (C.F.). |

# Reporting for specific materials, systems and methods

We require information from authors about some types of materials, experimental systems and methods used in many studies. Here, indicate whether each material, system or method listed is relevant to your study. If you are not sure if a list item applies to your research, read the appropriate section before selecting a response.

## Materials & experimental systems

| n/a | Involved in the study |
|---|---|
| ☒ | ☐ Antibodies |
| ☒ | ☐ Eukaryotic cell lines |
| ☒ | ☐ Palaeontology and archaeology |
| ☒ | ☐ Animals and other organisms |
| ☒ | ☐ Clinical data |
| ☒ | ☐ Dual use research of concern |
| ☒ | ☐ Plants |

## Methods

| n/a | Involved in the study |
|---|---|
| ☒ | ☐ ChIP-seq |
| ☒ | ☐ Flow cytometry |
| ☒ | ☐ MRI-based neuroimaging |

# Plants

| | |
|---|---|
| Seed stocks | *Report on the source of all seed stocks or other plant material used. If applicable, state the seed stock centre and catalogue number. If plant specimens were collected from the field, describe the collection location, date and sampling procedures.* |
| Novel plant genotypes | *Describe the methods by which all novel plant genotypes were produced. This includes those generated by transgenic approaches, gene editing, chemical/radiation-based mutagenesis and hybridization. For transgenic lines, describe the transformation method, the number of independent lines analyzed and the generation upon which experiments were performed. For gene-edited lines, describe the editor used, the endogenous sequence targeted for editing, the targeting guide RNA sequence (if applicable) and how the editor was applied.* |
| Authentication | *Describe any authentication procedures for each seed stock used or novel genotype generated. Describe any experiments used to assess the effect of a mutation and, where applicable, how potential secondary effects (e.g. second site T-DNA insertions, mosiacism, off-target gene editing) were examined.* |
